# Acute Chikungunya Infection Induces Vascular Dysfunction by Directly Disrupting Redox Signaling in Endothelial Cells

**DOI:** 10.3390/cells13211770

**Published:** 2024-10-25

**Authors:** José Teles de Oliveira-Neto, Juliano de P. Souza, Daniel Rodrigues, Mirele R. Machado, Juliano V. Alves, Paula R. Barros, Alecsander F. Bressan, Josiane F. Silva, Tiago J. Costa, Rafael M. Costa, Daniella Bonaventura, Eurico de Arruda-Neto, Rita C. Tostes, Emiliana P. Abrão

**Affiliations:** 1 Department of Pharmacology, Ribeirao Preto Medical School, University of Sao Paulo, Ribeirão Preto 14040-900, Brazil; 2 Department of Cellular and Molecular Biology and Pathogenic Bioagents, Ribeirao Preto Medical School, University of Sao Paulo, Ribeirão Preto 14040-900, Brazil; 3 Academic Unit of Health Sciences, Federal University of Jatai, Jataí 75804-068, Brazil; 4 Institute of Biomedical Sciences, University of Sao Paulo, Ribeirão Preto 05508-000, Brazil; 5 Department of Basic Health Sciences, Faculty of Medicine, Federal University of Mato Grosso, Cuiabá 79070-900, Brazil; 6 Institute of Biological Sciences, Federal University of Minas Gerais, Belo Horizonte 31270-901, Brazil; 7 Master’s Education Institute President Antonio Carlos (IMEPAC), Araguari 38025-440, Brazil

**Keywords:** Chikungunya, reactive oxygen species, nitric oxide synthase, vascular dysfunction

## Abstract

Chikungunya virus (CHIKV) infection is characterized by febrile illness, severe joint pain, myalgia, and cardiovascular complications. Given that CHIKV stimulates reactive oxygen species (ROS) and pro- and anti-inflammatory cytokines, events that disrupt vascular homeostasis, we hypothesized that CHIKV induces arterial dysfunction by directly impacting redox-related mechanisms in vascular cells. Wild-type (WT) and iNOS *knockout* (iNOS^−/−^) mice were administered either CHIKV (1.0 × 10^6^ PFU/µL) or Mock vehicle via the intracaudal route. In vivo, CHIKV infection induced vascular dysfunction (assessed by a wire myograph), decreased systolic blood pressure (tail-cuff plethysmography), increased IL-6 and IFN-γ, but not TNF-α levels (determined by ELISA), and increased protein content by Western blot. Marked contractile hyporesponsiveness to phenylephrine was observed 48 h post-infection, which was restored by endothelium removal. L-NAME, 1400W, Tiron, and iNOS gene deletion prevented phenylephrine hyporesponsiveness. CHIKV infection increased vascular nitrite concentration (Griess reaction) and superoxide anion (O_2_^•−^) generation (lucigenin chemiluminescence), and decreased hydrogen peroxide (H_2_O_2_, by Amplex Red) levels 48 h post-infection, alongside increased TBARS levels. In vitro, CHIKV infected endothelial cells (EA.hy926) and upregulated ICAM-1 and iNOS protein expression (determined by Western blot). These data support the conclusion that CHIKV-induced alterations in vascular ROS/NF-kB/iNOS/NO signaling potentially contribute to cardiovascular events associated with Chikungunya infection.

## 1. Introduction

The Chikungunya virus (CHIKV) is transmitted by hematophagous mosquitoes of the genus Aedes, frequently *Aedes (Stegomyia) aegypti* and *Aedes (Stegomyia) albopictus*, found mainly in tropical and subtropical regions, which can also transmit Dengue (DENV) and Zika (ZIKV) viruses. Although the most common arboviruses—Chikungunya, Dengue, and Zika—usually cause mild symptoms such as fever and arthralgia, cardiovascular complications are often reported, representing an increasing threat to urban and peri-urban populations outside of the tropical and subtropical areas in low- and middle-income countries [1,2,3].

Accordingly, we are currently witnessing a dramatic emergence/re-emergence of different arboviruses [4,5]. In addition to the annual epidemics caused by DENV in 2015 and 2016, there were severe ZIKV epidemics in South America in 2013 and 2014, and a Chikungunya epidemic quickly spread across the Caribbean and South America, posing new challenges to the public health system and emphasizing the need for multidisciplinary and innovative research [6,7].

In recent decades, CHIKV has become epidemic in several countries, causing disabling arthralgia and a wide spectrum of clinical manifestations, by mechanisms that are not yet completely elucidated [8]. CHIKV infection in its severe form is characterized by the development of arthralgia, and neurological and cardiovascular events [9,10], including symptoms of bradycardia, hypotension [11,12], shock, arrhythmias, myocarditis, and heart failure [9], as well as tachycardia and cardiac arrest [2].

The inflammatory process, or the sequence of events coordinated by the immune system following CHIKV infection, involves complex interactions between inflammatory cells, such as neutrophils, lymphocytes, and monocytes/macrophages, as well as vascular cells [13,14]. Such interactions result in increased tissue production of soluble mediators as well as complement system proteins, chemokines, cytokines, reactive oxygen species (ROS), vasoactive amines, and increased expression of cell adhesion molecules in circulating leukocytes and endothelial cells, among others [15].

Endogenous sources of reactive oxygen species (ROS) include NADPH oxidase enzymes (NOXs), mitochondria, endoplasmic reticulum, and peroxisomes. Superoxide dismutase 1 (SOD1) rapidly converts cytosolic superoxide (O_2_^•−^) to hydrogen peroxide (H_2_O_2_). H_2_O_2_ oxidizes key thiols in proteins to control a variety of biological processes, including metabolic adaptability, differentiation, and proliferation. H_2_O_2_ can combine with metal cations (Fe_2_^+^ or Cu^+^) to form the hydroxyl radical (OH^−^), resulting in irreversible oxidative damage to lipids, proteins, and DNA. ROS signaling and oxidative stress are frequently connected with endothelial dysfunction, which plays a role in the pathophysiology of numerous vascular-related disorders [16].

The controlled generation of ROS activates signaling pathways involved in normal endothelial activities, including angiogenesis. Redox signaling processes in the cardiovascular system rely on precise and reversible changes in target proteins caused by ROS or reactive nitrogen species (RNS), which control endothelial function and the development of vascular disorders. The redox balance in the vascular endothelium is disrupted under a variety of clinical situations, and it plays an important role in the etiology of vascular disorders [17].

Cytokines produced by immune cells trigger and regulate the recruitment of neutrophils and monocytes to the injury site [18], increase vascular permeability, and stimulate ROS generation and the overproduction of nitric oxide (NO) [19,20]. In viral infections, NO and peroxynitrite (ONOO^–^), formed by the reaction between NO and superoxide anion (O_2_^•−^), are key molecules in the human primary immune defense, with direct and indirect antiviral activity [21]. Direct antiviral activity, for example, is based on the ability of NO to inactivate viral particles or inhibit their replication, while indirect activity consists of modulating the host’s immune response that generates an inflammatory response [22].

While NO and ONOO^–^ have direct antiviral effects on the pathogen, they also participate in oxidative stress, which causes severe cytotoxic effects as well as non-specific oxidative damage in infected tissues, including the vasculature. More specifically, NO causes the S-nitrosylation of proteins, and ONOO^–^ causes the nitration of proteins, lipids, and DNA [23]. The oxidative damage induced by ONOO^–^ inactivates these biomolecules, triggering degradation pathways and even cell death events [23]. Although the vasculature is a key component in this series of events, the deleterious effects of a virus infection are usually attributed to immune cell-related mechanisms.

In this study, we tested the hypothesis that CHIKV infection of endothelial cells leads to oxidative stress and altered NO production and signaling, and that these are key events for vascular abnormalities in CHIKV infection. Here, we determined whether CHIKV interferes with endothelial cell function and the mechanisms that lead to CHIKV effects.

## 2. Materials and Methods

### 2.1. Animals

All experimental protocols were performed in accordance with the National Council for Animal Experimentation Control and were approved by the Ethics Committee on Animal Use of the University of Sao Paulo, Ribeirao Preto, Brazil (Protocol n° 171/2019). Male 6-week-old C57BL/6J wild-type (WT) and iNOS *knockout* (iNOS^−/−^) mice were obtained from the Isogenic Breeding Unit at the Ribeirao Preto Medical School, University of Sao Paulo, Ribeirao Preto, Brazil. Mice were maintained in a room with controlled temperature (22 ± 1 °C) and humidity (50–60%), on a 12 h light/dark cycle with ad libitum access to food and water. The Chikungunya viral stock (CHIKV) used in the in vivo and in vitro assays was obtained from Prof. Eurico de Arruda-Neto. After 1 week of acclimatization, mice were administered either CHIKV (1.0 × 10^6^ PFU/µL) or Mock vehicle (used for viral replication) via the intracaudal route. Mice were divided into four experimental groups: (1) WT_Mock; (2) WT_CHIKV; (3) iNOS^−/−^_Mock; and (4) iNOS^−/−^_CHIKV. Mice were euthanized at three time points: 24, 48, and 72 h following infection (n = 4–6 animals per group).

### 2.2. Blood Pressure Measurement

Systolic blood pressure (SBP) was determined by an indirect method, using the tail-cuff plethysmography method. The transduction system (CODA V.1.1, Kent Scientific, Torrington, CT, USA) was connected to an electric sphygmomanometer via an adjustable occluder and sensor on the proximal part of the mouse’s tail. The mice were put in a restraining cylinder with apertures for their nose and tail after being warmed for ten minutes (min) at 38 °C. The average of three consecutive readings, expressed in millimeters of mercury (mmHg), is the final blood pressure reading for every mouse. Before the blood pressure readings, mice were allowed to get used to the apparatus and procedures for five days.

### 2.3. Determination of Cytokine Levels by Enzyme-Linked Immunosorbent Assay

Serum interleukin-6 (IL-6), interferon-gamma (IFN-γ), and tumor necrosis factor-alpha (TNF-α), all related to activation of the NF-κB pathway, were used as biomarkers for severe infection caused by CHIKV and were quantified by Enzyme-Linked Immunosorbent Assay [(ELISA) R&D Systems, MLB00C]. Serum cytokines were measured in infected (CHIKV 1 × 10^6^ PFU/µL) and Mock-treated mice.

### 2.4. Functional Vascular Studies

After anesthesia with isoflurane and euthanasia, thoracic aortas were isolated and placed in a modified Krebs–Henseleit nutrient solution [(composition in mol/L): NaCl, 130; KCl, 4.7; KH_2_PO_4_, 1.18; MgSO_4_, 1.17; NaHCO_3_, 14.9; EDTA, 0.026; CaCl_2_.2H_2_O, 1.56; glucose, 5.5] at 4 °C. The median portion of the thoracic aorta was divided into 4 rings of 2 mm each. Endothelium-intact rings were mounted on a myograph (model 620M; Danish Myo Technology–DMT, Copenhagen, Denmark), and isometric force generation was recorded using a signal transducer (ML T001 isometric voltage transducer, Power Lab/8S, AD Instruments Pty Ltd. New South Wales, Australia) coupled to a computer. The myograph vats contained modified Krebs–Henseleit solution aerated with 95% O_2_ and 5% CO_2_, and heated to 37 °C. The preparations remained under 5 mN of tension for 30 min (min) for stabilization, with changes in nutrient solution and tension adjustment every 10 min.

After the stabilization period, the integrity of the endothelium was tested by assessing relaxation to acetylcholine (ACh, 10^−6^ mol/L, a muscarinic receptor agonist that induces endothelium-dependent vasodilation) in aortic rings constricted with phenylephrine (PE, 10^−7^ mol/L, an alpha-adrenergic agonist). Concentration–effect curves for PE and ACh (10^−11^ mol/L–10^−5^ mol/L) were performed in endothelium-intact and endothelium-denuded vascular segments.

To determine whether nitric oxide synthase (NOS) enzymes contribute to vascular dysfunction induced by CHIKV infection, vessels were incubated with the non-selective NOS inhibitor L-NAME (10^−4^ mol/L) and the selective inhibitor from iNOS, 1400W (10^−6^ mol/L). To test whether the vascular changes induced by CHIKV infection depend on the generation of ROS, the vessels were incubated with Tiron (10^−4^ mol/L–selective O_2_^•−^). In functional vascular studies with inhibitors, aortic rings were incubated for 30 min. For each concentration–effect curve, the maximum response and pEC_50_ were calculated using nonlinear regression analysis. 

### 2.5. Cultured Endothelial Cells’ Viability, Viral Replication Profile, and Quantification

Immortalized human vascular endothelial cells (EA.hy926) were obtained from the Rio de Janeiro Cell Bank (BCRJ–code 0345) and infected with CHIKV, using different multiplicities of infection (MOIs) and different time points. Endothelial cells were maintained at 37 °C in 75 cm^2^ flasks (Corning, New York, NY, USA) containing DMEM (Invitrogen, Waltham, MA, USA), supplemented with 10% fetal bovine serum (FBS), 1% L–200 mM glutamine, 1% antibiotic (100 U/mL penicillin, 1 mg/mL streptomycin) (Invitrogen), and 10% Tryptose, in an environment with 5% CO_2_.

To assess viral replicative capacity in endothelial cells, assays to quantify the replication profile were performed. After infections with different MOIs and at different time points, daily collections of the cell’s supernatant were performed. The supernatants, with the viral progenies released after the viral replicative cycle, were aliquoted in microtubes and stored at −80 °C. The CHIKV genomic RNAs were extracted from the supernatants using a kit for viral RNA extraction (Qiagen, Hilden, Germany), with subsequent quantification by real-time RT-PCR (reverse transcription polymerase chain reaction) using the QuantiTect SYBR Green RT-PCR Kit (Qiagen) and the primers CTCATACCGCATCCGCATCAG (forward) and ACATTGGCCCCACAATGAATTTG (reverse).

The graph was generated using the parameter “ct”, or threshold cycle, which corresponds to the number of the PCR cycle in which the sample reaction curve intersects the threshold line. The ct value indicates how many cycles are needed to detect a real signal from the samples, with a small ct indicating a high concentration of genetic material.

Cell viability after endothelial cell infection with MOIs (multiplicity of infection–ratio of CHIKV to EA.hy926 cells) of 0.5, 1.0, and 2.0 was determined by quantifying the reduction in 3-(4,5-dimethylthiazol-2-yl)-2.5 bromide of diphenyl tetrazolium (MTT, Sigma-Aldrich), as previously described by Mosmann (1983) [24]. In 96-well plates, 3.0 × 10^4^ cells per well were plated and incubated in a CO_2_ incubator (5%) at 37 °C, overnight. Infections were performed using a 1.0 MOI for 48 h (h).

After establishing the infection time and MOI, EA.hy926 cells were cultured using Dulbecco’s Modified Eagle Medium (DMEM, Thermo Fisher Scientific, Waltham, MA, USA), and maintained in an incubator at 37 °C and 5% CO_2_. Cells at ~80% confluence were infected with different multiplicities of infection of CHIKV or were exposed to vehicle (Mock). Cells were then washed, harvested in lysis buffer, and kept frozen until further use.

### 2.6. Quantification of NO Production

Cells were cultured and exposed to Mock or CHIKV (MOI 1.0) for 48 h. Cells were then washed with 1X PBS and incubated for 1 h with the fluorescent probe 4,5-diaminofluorescein (5 µmol/L; Sigma-Aldrich, St. Louis, MO, USA; catalog no. 50277). Medium fluorescence was measured at room temperature using a multi-mode microplate reader (FlexStation 3, Molecular Devices, San Jose, CA, USA) with excitation and emission wavelengths of 491 nm and 513 nm, respectively. NO levels are expressed by the diaminofluorescein (DAF) fluorescence intensity, and changes in NO production were calculated as a percentage of control values (% of Baseline levels).

### 2.7. Griess Reaction

NO production was indirectly measured via the concentration of NO_2_^−^ (nitrite) in the serum of WT_Mock and WT_CHIKV mice and in the endothelial cell’s supernatant, in the presence of nitrate reductase and in the absence of light, at 37 °C, using the Griess colorimetric assay, with subsequent reading in a spectrophotometer at 540 nm. The serum concentrations of nitrates were obtained by subtracting the nitrite values from the NOx values (nitrite + nitrate).

### 2.8. Lucigenin Chemiluminescence

The generation of superoxide anion (O_2_^•−^) in thoracic aortas was measured by chemiluminescence assay, using lucigenin as an electron acceptor of nicotinamide, and adenine dinucleotide (NADH) as a substrate. Mouse thoracic aortas infected with CHIKV (1 × 10^6^ PFU and MOI 1, respectively) or vehicle (Mock) were washed and harvested in ROS buffer/Lysis buffer. NADPH (10^−4^ mol/L) was added to the suspension containing lucigenin (5 μM). Luminescence was measured before and after stimulation with NADPH. Values from a buffer blank were subtracted from each reading. The results are expressed as a fold change in arbitrary units per milligram of protein (measured by the BCA assay).

### 2.9. Measurement of Hydrogen Peroxide (H_2_O_2_) Production by Amplex Red

H_2_O_2_ production was evaluated in 50 µL aliquots of tissue extracts and supernatant from EA.hy 926 from the Mock- and CHIKV-infected groups, using a H_2_O_2_ measurement kit, Amplex Red (Molecular Probes, Invitrogen). Emitted fluorescence was measured at an excitation wavelength of 530 nm and an emission wavelength of 590 nm. The standard curve for H_2_O_2_ was constructed using the manufacturer’s standards, at room temperature, and used to determine the H_2_O_2_ concentration of the samples. The result was expressed as the average of the absolute concentrations of H_2_O_2_ detected in the samples (µM) corrected by total proteins.

### 2.10. Assessment of Levels of Oxidative Stress Markers

One of the mechanisms of injury in oxidative stress is the oxidation of the lipid layer of the cell membrane, a process known as lipoperoxidation (LPO). The by-products arising from this LPO can be detected by an assay that uses thiobarbituric acid as a reagent, through the technique known as TBARS (thiobarbituric acid reactive substances). Therefore, we evaluated whether CHIKV infection in endothelial cells (EA.hy 926) favors the generation of these markers of oxidative stress with changes in the levels of substances reactive to thiobarbituric acid (TBARS).

### 2.11. Western Blot

The protein contents of iNOS, ICAM-1, NF-κB p65, (p)-NF-κB-p65^Ser536^, and β-actin were determined by Western blot analysis in samples from cultured endothelial cells and isolated aortas. Samples were homogenized in lysis buffer, and proteins were kept frozen at −80°C until use. Proteins (30 μg) were separated by electrophoresis on 10 or 12% polyacrylamide gels, transferred to 0.22 μm nitrocellulose membranes, and blocked using 5% bovine serum albumin (BSA) in Tris-buffered saline (TBS) and 0.1% Tween 20 for 1 h. Primary antibodies were incubated overnight at 4 °C as follows: anti-phospho p65 [(p)-NF-κB-p65^Ser536^] (1:1000; Cell Signaling 3033); anti-NF-κB p65 (1:1000; Cell Signaling 8242); anti-iNOS (1:1000; Sigma-Aldrich SAB4502011); anti-ICAM-1 (1:500; Santa Cruz Biotechnology sc-8439); and anti-β-actin-peroxidase (1:15000; Sigma-Aldrich A3854). Protein bands were detected by chemiluminescence reaction (Luminata Forte, WBLUF0100, Merck-Millipore, Watford, UK), and the intensity of the bands was evaluated by densitometric analysis using the ImageQuant^TM^ LAS 4000 software version 1.2.

### 2.12. Statistical Analysis

For the analysis of vascular reactivity, maximum response and pEC_50_ (negative logarithm of the EC_50_) values were determined from the concentration–response curves that were fit using nonlinear curve fitting. Area under the curve (AUC), Emax, and pEC_50_ values were compared using Student’s *t*-test, One-way ANOVA and Two-way ANOVA tests, followed by the Dunnett and Tukey post-tests, respectively. The results of the molecular experiments were analyzed by Student’s *t*-test or One-way ANOVA, followed by the Tukey post-test. The (GraphPad Prism version 10.0.0 for Windows, GraphPad Software, Boston, MA, USA, www.graphpad.com), was used for data analysis. The results are expressed as mean ± standard error of the mean (SEM). The acceptable level of significance was *p* < 0.05.

## 3. Results

### 3.1. CHIKV Infection Induces Vascular Dysfunction

**Figure 1:**
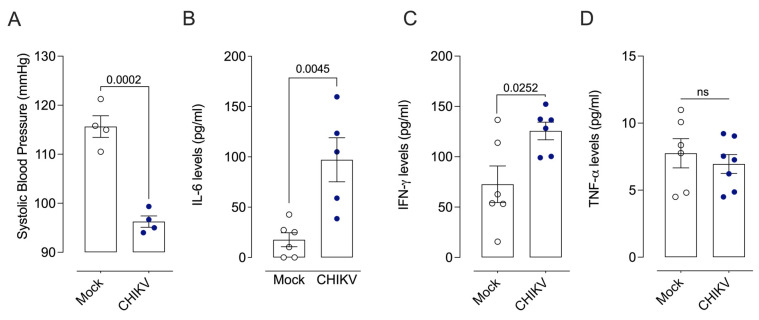
Characterization of the animal models infected by Chikungunya (CHIKV). CHIKV decreases systolic blood pressure and increases circulating cytokine levels in mice 48 h post-infection. (**A**): Measurement of systolic blood pressure (mmHg); (**B**–**D**): IL-6, IFN-γ, and TNF-α levels in the serum of mice 48 h after Mock (open white circles) or CHIKV (1.0 × 10^6^ PFU/µL) (solid blue circles) administration. Data are expressed as mean ± S.E.M. *p* < 0.05 vs. WT_Mock; Student’s *t*-test was used for statistical analysis; *n* = 4–7 per group. WT: wild type–wild mice.

**Figure 2:**
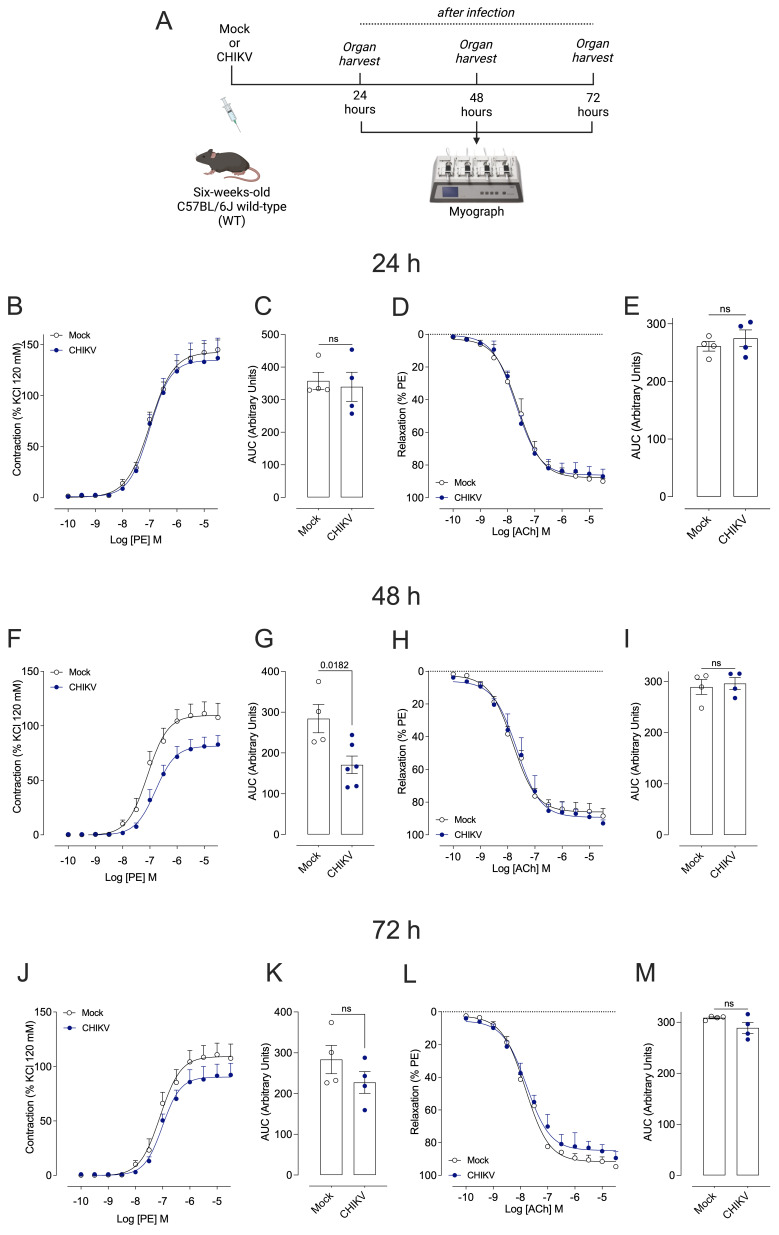
Cumulative concentration–effect curves for phenylephrine (PE) and acetylcholine (ACh) in endothelium-intact aortas from Mock-treated and CHIKV-infected mice. (**A**): Representative figure of the experimental design. (**B**,**F**,**J**): Vascular responses to PE—24, 48, and 72 h after Mock (open white circles) or CHIKV (1.0 × 10^6^ PFU/µL) (solid blue circles) administration, respectively. (**D**,**H**,**L**): Vascular responses to Ach—24, 48, and 72 h after Mock (open white circles) or CHIKV (1.0 × 10^6^ PFU/µL) (solid blue circles) administration, respectively. (**C**,**E**,**G**,**I**,**K**,**M**): The bar graphs show the area under the curve (AUC) in the concentration–effect curves. Data are expressed as mean ± S.E.M. *p* < 0.05 vs. WT_Mock; Student’s *t* test was used for statistical analysis; *n* = 4–6 animals per group.

Forty-eight (48) hours after infection, SBP was lower in animals infected with CHIKV when compared to the control group (Mock) (Figure 1A). IL-6 (Figure 1B) and IFN-γ (Figure 1C) levels, but not TNF-α (Figure 1D), were increased in CHIKV-infected mice. Marked contractile hyporesponsiveness to phenylephrine was observed at 48 h (Figure 2F,G) but not at 24 h (Figure 2B,C) or 72 h (Figure 2J,K) post-CHIKV infection. Although the contractile response remained unchanged 72 h (Figure 2J) after infection, there was a difference in the maximum response to PE (Table 1). No changes in acetylcholine-induced vasodilation were observed at any of the time points (Figure 2D,E,H,I,L,M). In addition, infection with CHIKV or Mock vehicle did not affect the body mass (g) of WT and iNOS^−/−^ mice (Table S1).

### 3.2. The Endothelium Drives the Vascular Changes in CHIKV-Infected Mice

**Figure 3:**
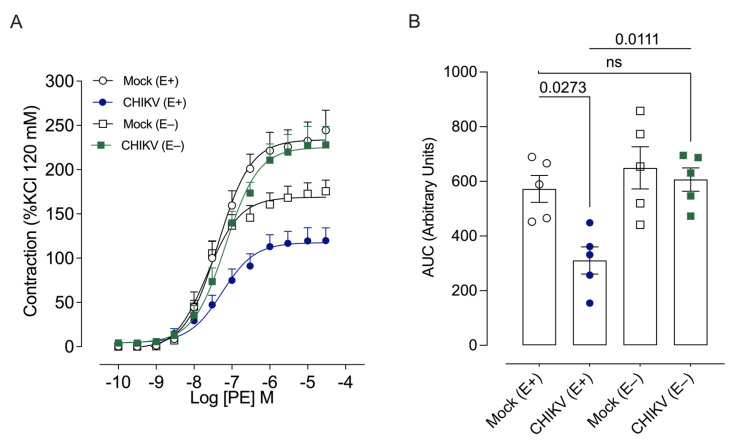
Cumulative concentration–effect curves for phenylephrine (PE) in endothelium-intact and endothelium-denuded aortic rings from Mock-treated and CHIKV-infected mice. (**A**): Constrictor responses to PE in arteries with or without intact endothelium, 48 h after Mock (open white circles and squares) or CHIKV (1.0 × 10^6^ PFU/µL) (solid blue circles, and solid green squares) administration. (**B**): The bar graph shows the area under the curve (AUC) in the concentration–effect curves. Data are expressed as mean ± S.E.M. *p* < 0.05 vs. WT_Mock; One-way ANOVA test, followed by Dunnett post-test; *n* = 5–6 animals per group. E+: with endothelium; E–: without endothelium.

Based on these results, the time of 48 h was chosen for subsequent evaluations of the mechanisms that mediate vascular dysfunction in CHIKV-infected mice. Vascular reactivity assays performed in endothelium-denuded aortic rings 48 h post-infection showed that removal of the endothelium restored vasoconstrictor responses to PE in CHIKV-infected mice and abolished the differences between the groups (Figure 3).

### 3.3. Pharmacological Inhibition and Deletion of iNOS Prevent In Vivo Effects of CHIKV Infection on Vascular Function

**Figure 4:**
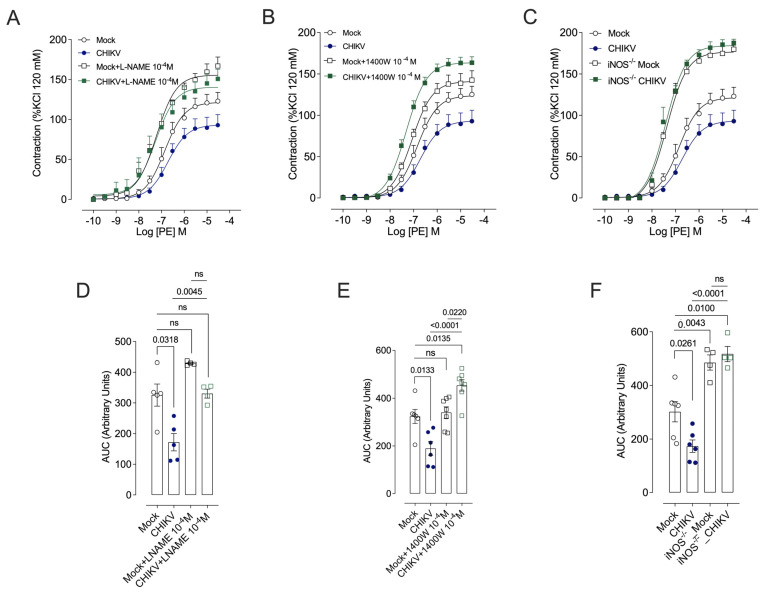
Cumulative concentration–effect curves for phenylephrine (PE) in the presence of pharmacological inhibitors (L-NAME and 1400W) and in iNOS ^–/–^ mice. (**A**–**C**): Vasoconstrictor responses to PE were determined in the presence of vehicle, L-NAME 10^–4^ M, or 1400W 10^–4^ M, and in iNOS^–/–^ mice, 48 h after Mock (open white circles and squares) or CHIKV (1.0 × 10^6^ PFU/µL) (solid blue circles, and solid green squares) administration. (**D**–**F**): The bar graphs depict the area under the curve (AUC) for the concentration–effect curves performed in vessels exposed to vehicle, L-NAME 10^–4^ M, and 1400W 10^–4^ M, and from iNOS^–/–^ mice. Data are expressed as mean ± S.E.M. *p* < 0.05 vs. WT_Mock; Two-way ANOVA test, followed by Tukey’s post-test, was used for statistical analysis; *n* = 4–6 animals per group.

Both L-NAME (Figure 4A,D) and 1400W (Figure 4B,E) abolished differences in the contractile response to PE between aortic rings from Mock-treated and CHIKV-infected mice. However, the aortic rings of infected animals incubated with 1400W showed a greater contractile response than those obtained from Mock animals with 1400W. Furthermore, iNOS gene deletion prevented PE hyporesponsiveness induced by CHIKV infection (Figure 4C,F). Incubation with L-NAME, 1400W, and gene deletion (iNOS^−/−^) raises pEC50, while Tiron shows no effect, implying a decrease in PE potency compared to Mock. In addition, incubation with NOS inhibitors leads to a greater pEC50 than in the CHIKV group (Table 2).

### 3.4. CHIKV Infection Increases Vascular NO Production, Reactive Oxygen Species Generation, and Causes Oxidative Stress

**Figure 5:**
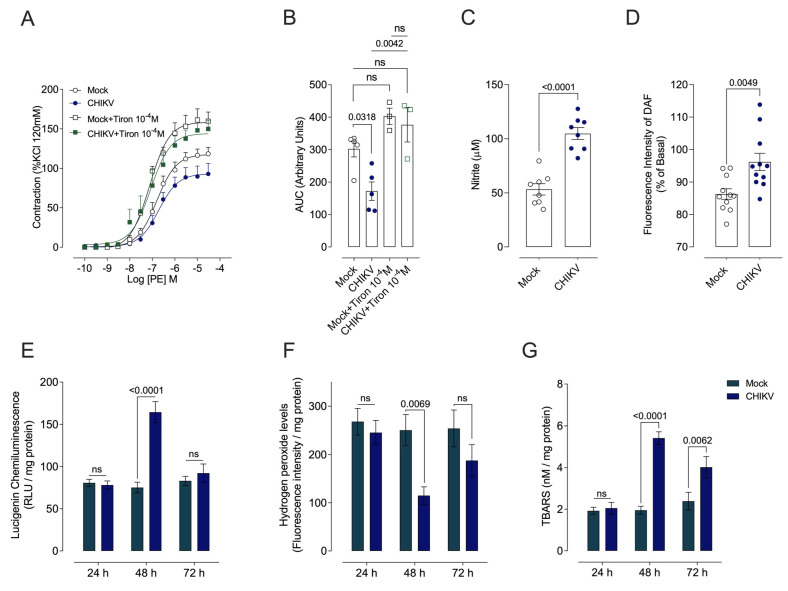
CHIKV infection increases vascular superoxide anion (O_2_^•–^) generation and oxidative stress markers in vascular cells, and serum nitrite levels. (**A**): Vasoconstrictor responses to PE were determined in the presence of vehicle or Tiron 10^–4^ M. (**B**): The bar graphs depict the area under the curve (AUC) for the concentration–effect curves in vessels exposed to vehicle or Tiron 10^–4^ M. (**C**): NO concentration (indirect measurement of nitrite levels). (**D**): NO levels (direct measurement of DAF fluorescence intensity). The graphics show levels of (**E**): superoxide anion; (**F**): oxidative stress markers; and (**G**): thiobarbituric acid reactive species, 48 h after Mock (open white circles and squares) or CHIKV (1.0 × 10^6^ PFU/µL) (solid blue circles, and solid green squares) administration. Data are expressed as mean ± S.E.M. *p* < 0.05 vs. WT_Mock; Two-way ANOVA test, followed by Tukey’s post-test or Student’s *t* test, was used for statistical analysis, *p* < 0.05 vs. Mock; *n* = 3–8 animals per group.

The variations in the contractile responses of aortic rings to PE between Mock- and CHIKV-infected mice were eliminated by Tiron (Figure 5A,B). CHIKV infection at 48 h increased nitrite concentration (indirect measurement of NO) (Figure 5C), NO levels (direct measurement of NO by fluorescence intensity of DAF) (Figure 5D), and O_2_^•−^ generation (Figure 5E), and decreased H_2_O_2_ (Figure 5F). CHIKV also increased levels of TBARS 48 h post-CHIKV infection (Figure 5G). No changes in O_2_^•−^ or H_2_O_2_ were detected 24 h or 72 h (Figure 5E,F) post-CHIKV infection, but TBARS levels remained increased 72 h post-infection (Figure 5G). 

### 3.5. Endothelial Cells’ Infection and Viability, Viral Replication Profile, and Quantification

**Figure 6:**
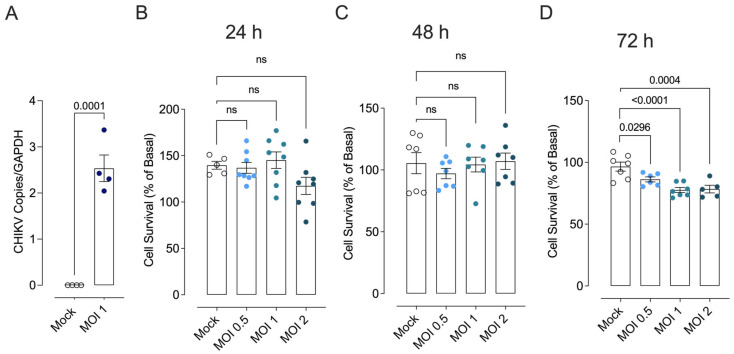
CHIKV replication profile in endothelial cells and cell viability. (**A**): Cells were infected with MOI 1 and viral replication was evaluated in the cell supernatant 48 h post-infection. (**B**–**D**): Cell viability determined under different MOIs [0.5 (solid light blue circles), 1 (solid light green circles), and 2 (solid dark green circles)] and time points (24, 48, and 72 h) after Mock (open white circles) or CHIKV infection. Data are expressed as mean ± S.E.M. *p* < 0.05 vs. WT_Mock; Student’s *t* test and Two-way ANOVA test, followed by Tukey’s post-test, were used for statistical analysis; *n* = 5–8 per group.

To determine whether CHIKV infects endothelial cells and directly leads to endothelial dysfunction, the ability of CHIKV to replicate in endothelial cells was verified. Viral infection was consistently detected with an MOI of 1 (Figure 6A), showing that endothelial cells are permissive to viral infection. A MOI of 2 decreased cell viability at all times evaluated (Figure 6B–D). Thus, the in vitro assays were carried out with an MOI of 1 in line with the replication test previously carried out. 

### 3.6. CHIKV Infection Activates NF-κB Signaling in Vascular Cells In Vivo and In Vitro

**Figure 7:**
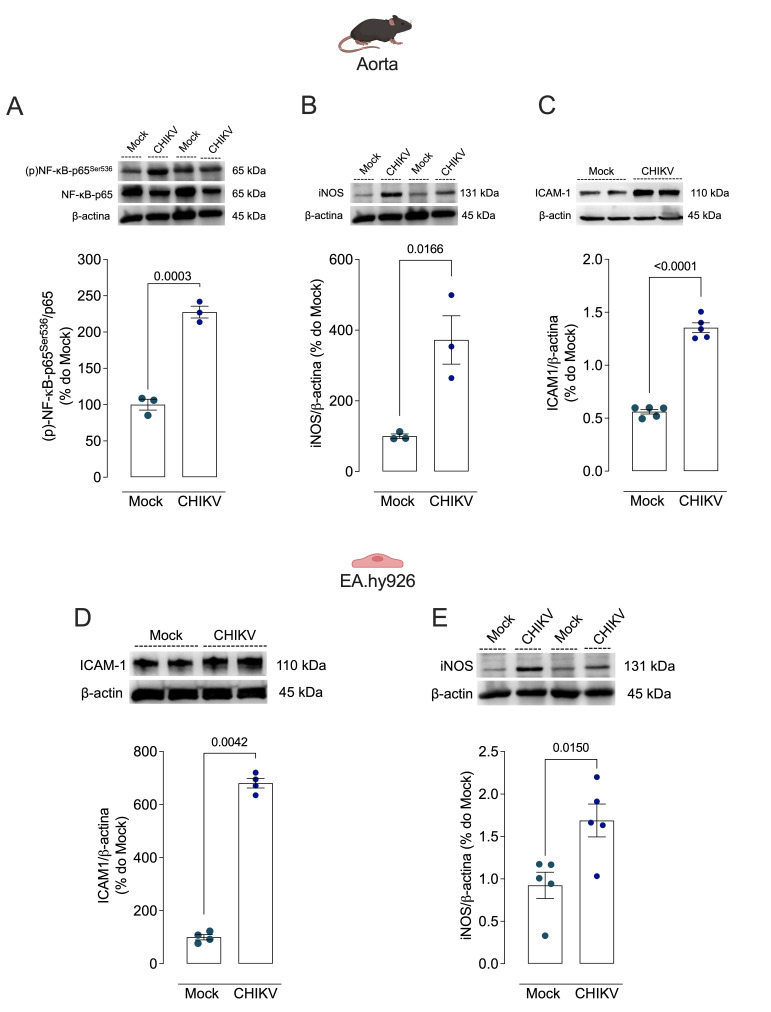
Infection with CHIKV increases activation of vascular NF-κB signaling. (**A**–**C**): Vascular expression of proteins (p)-p65Ser536/total NF-κB p65 ratio, iNOS, and ICAM-1, respectively. (**D**,**E**): ICAM-1 and iNOS protein expression in endothelial cells, 48 h after Mock (solid light blue circles) or CHIKV (1.0 × 10^6^ PFU/µL) (solid blue circles) administration. Protein expression was determined by Western blot. Data are expressed as mean ± S.E.M. *p* < 0.05 vs. WT_Mock; Student’s *t* test was used for statistical analysis; *n* = 3–5 per group.

To address NF-κB activation in aortas from CHIKV-infected mice, the expression of the total and phosphorylated forms of p65 was assessed. Aortas of the WT_CHIKV mice exhibited increased expression of the proteins (p)-NF-κB p65^Ser536^/p65 total ratio (Figure 7A), iNOS (Figure 7B), and ICAM-1 (Figure 7C). In endothelial cells, CHIKV infection increased ICAM-1 (Figure 7D) and iNOS protein expression (Figure 7E).

## 4. Discussion

Our study focuses on vascular dysfunction brought on by CHIKV infection. The present study shows that (i) the endothelium plays a fundamental role in driving vascular changes in CHIKV-infected mice, (ii) CHIKV infection directly impacts NO production, vascular reactive oxygen species, and oxidative stress, and (iii) CHIKV infection activates NF-κB signaling in vascular cells. Our findings clearly show that CHIKV infection induces vascular dysfunction and decreases SBP in mice.

These results are in line with earlier studies suggesting that infection by other arboviruses, and even other types of viruses, might negatively impact vascular function. Key mechanisms by which DENV impairs vascular function include the disruption of the endothelial glycocalyx, the activation of matrix metalloproteinases, and the alteration of inter-endothelial junctions. All these processes work together to cause severe clinical symptoms like hypovolemic shock and increased vascular leakage [25,26].

Intranasal infection with the betacoronavirus Mouse Hepatitis Virus Type 3 (MHV-3), for example, leads to significant vascular alterations—a marked reduction in vascular contractility in the aorta and vena cava—accompanied by severe hypotension and decreased peripheral blood flow [27]. Compared to other viruses like DENV and ZIKV, CHIKV also affects vascular function and cardiovascular health in a similar way. All three viruses have the propensity to cause major inflammatory reactions and endothelial dysfunction, which can result in increased permeability and vascular problems [28]. The necessity of closely monitoring and treating cardiovascular problems during virus outbreaks is highlighted by these common mechanisms.

Vascular hyporesponsiveness, which may contribute to hypotension and decreased blood flow in both CHIKV and MHV-3 [27], suggests a complex hemodynamic response in viral infections. Marked contractile hyporesponsiveness to PE observed mainly 48 h post-infection relies on endothelial cell dysfunction, in contrast to sepsis-induced vasoplegia, which occurs due to a combination of endothelial and smooth muscle dysfunction [22].

The increased vascular production of IL-6 and IFN found in the present study, as well as the increased TNF production induced by MHV-3 infection [27], may represent endothelial responses to the systemic inflammatory process provoked by CHIKV infection. However, these effects may also result from direct viral infection of endothelial cells. Microvascular endothelial cells are susceptible to SARS-CoV-2 infection, contributing to viral amplification but triggering pro-inflammatory mediators, which is consistent with clinical investigations showing endotheliitis and organ damage in severe COVID-19 patients [27,29].

The endothelium closely controls vascular tone, immune responses, coagulation, permeability, and blood fluidity [30,31]. Phenotypic alterations brought on by endothelial dysfunction include prothrombotic states, increased cytokine and adhesion molecule levels, and increased vascular contractility and permeability [32] CHIKV infection disrupts these events, emphasizing how crucial it is to understand endothelial (dys)function in the pathogenesis of CHIKV infection.

MHV-3 infection decreases the contractility of the aorta and vena cava, a phenomenon that can be restored to normal levels by interventions that include removal of the endothelium, inhibition of iNOS, genetic deletion of iNOS, or NO elimination, highlighting the importance of endothelial function, with iNOS and NO playing key mediating roles in this process, in vascular regulation during MHV-3 infection [27].

Similarly, this study shows that removal of the endothelium restores vasoconstrictor responses to phenylephrine (PE) and eliminates disparities between CHIKV-infected and un-infected groups. Furthermore, both L-NAME and 1400W nullify the differences in the contractile response to PE between aortic rings of Mock-treated and CHIKV-infected mice. In addition, genetic deletion of iNOS prevents PE hyporesponsiveness induced by CHIKV infection.

In general, excessive NO reduces NO sensitivity and restricts blood vessel responsiveness to vasodilators [33]. The increased NO production induced by CHIKV infection, i.e., the higher concentration of nitrate in infected mice, is key to vascular dysfunction, since overproduction of NO decreases vascular contractility [34]. For example, vasoplegia and hypotension observed in sepsis result from an excess of NO [22,35].

ROS are important elements of antimicrobial cell defense [19], and CHIKV infection increased superoxide anion (O_2_^•−^) levels in mouse-isolated aortas. Additionally, pharmacological and molecular suppression of NOS activity confirmed the involvement of iNOS and ROS in CHIKV-induced vascular dysfunction.

Excessive ROS generation may be an additional factor in the vascular alterations and endothelial dysfunction linked to cardiovascular illness [36,37,38] in the context of CHIKV infection. These findings point to a common mechanism involved in the control of blood pressure and vascular contractility during viral infections.

In the in vitro experiments, infection of endothelial cells with CHIKV also increased O_2_^•−^ generation and (p)-NF-κB subunit expression. As ROS are related to cell activation, they can facilitate viral replication, depending on the cell and the virus types [39]. Accordingly, MHV-3 infection induces vascular dysfunction through the production of iNOS-derived NO, the generation of ROS, and the activation of the NF-κB pathway, and decreases blood pressure [27].

NF-kB is a protein complex involved in gene transcription, activation of survival enzymes, generation of inflammatory cytokines, and oxidative processes [40,41]. An increased (p)-NF-κB p65^Ser536^/p65 total ratio, which indicates activation of NF-kB signaling, was found in arteries of animals infected with CHIKV [41,42]. Ser^536^ phosphorylation of the p65 subunit contributes to the increased efficiency of NF-κB translocation to the nucleus [43].

Phosphorylation of NF-κB p65^Ser536^ regulates the interaction with corepressor and coactivator factors, thus directly contributing to the increase in gene transactivation [44], which could explain the increased iNOS and ICAM-1 expression in the arteries of the infected animals. Furthermore, increased ICAM-1 and iNOS were also found in endothelial cells in vitro, reinforcing the direct actions of CHIKV in vascular cells.

The findings regarding increased ICAM-1 expression in endothelial cells in vitro further underscore the direct impact of pathogens like CHIKV on endothelial dysfunction. ICAM-1 and VCAM-1’s interaction with NF-kB activation assumes crucial significance in this context, particularly in cardiovascular diseases, where heightened levels of these adhesion molecules exacerbate vascular inflammation and dysfunction. As pro-inflammatory cytokines drive the upregulation of soluble VCAM-1, leukocyte transmigration and subsequent accumulation on vessel walls are facilitated, culminating in vascular narrowing and the development of cardiovascular dysfunction [45].

Furthermore, the heightened NF-kB activity facilitated by elevated ICAM-1 and VCAM-1 levels in the bloodstream amplifies inflammatory pathways, fostering cardiac remodeling and fibrosis. This intricate interplay underscores the pivotal roles of ICAM-1 and VCAM-1 in cardiovascular pathophysiology and accentuates their potential as therapeutic targets for mitigating cardiovascular disease progression, particularly through the modulation of NF-kB signaling [46].

Ser^276^ phosphorylation in RelA, another component of the NF-κB complex, has been shown to be ROS-dependent [41]. During Dengue infection, there is cytokine-driven ROS generation in endothelial cells, which reinforces our finding that CHIKV infection causes NF-κB-mediated inflammatory processes in the vasculature [39,47]. ROS can interact with polyunsaturated fatty acids and initiate lipid peroxidation, a process that leads to a loss of membrane function and integrity. Furthermore, increased vascular permeability observed in Dengue Hemorrhagic Fever is related to endothelial cell dysfunction and not to the structural destruction of endothelial cells [47,48].

In line with our findings, in an experimental model of influenza virus infection, exudated macrophages and bronchial epithelial cells in lung tissues of infected mice express that iNOS and NO production is completely blocked by the pharmacological inhibition of NOS with Nω-monomethyl-l- arginine (L-NMMA) and by the genetic deletion of iNOS, indicating that the excessive production of NO is due to the expression of iNOS in areas of viral infection [49]. Accordingly, increased iNOS expression was observed in the aorta of CHIKV-infected mice. It is possible that iNOS increases occur in the endothelium, since endothelium removal restored the vascular responses.

Pathological results also highlight how crucial it is to investigate how viral infections affect the cardiovascular system. Reports have shown the frequency of cardiac involvement in arbovirus infections [50,51,52]. In CHIKV infections, cardiovascular failure in atypical and severe forms have been demonstrated. Low incidence of myocarditis has also been demonstrated, as well as comorbidities in more than 90% of cases, such as with hypertension, diabetes mellitus, kidney disease, ischemic heart disease, and chronic heart disease [2]. Regarding Zika virus infection, a retrospective analysis of newborns in northeastern Brazil showed that more than 13% of congenital cardiac abnormalities were related to infection [53].

Biomarkers linked to endothelial and cardiac dysfunction were shown to be markedly increased in patients with deadly Chikungunya virus (CHIKV) infections. Examining these biomarkers could help predict the clinical course and advance our knowledge of the pathophysiology of deadly CHIKV infections [54]. Additionally, the most common atypical presentation is cardiovascular (CV) involvement, which occurs in 54.2% of cases. Although the expected CV mortality rate is 10%, patients with comorbidities may have a 20% CV mortality rate [55]. Thus, the confluence of CHIKV-related cardiovascular symptoms and indicators emphasizes the necessity of prompt diagnosis and suitable treatment to enhance patient outcomes.

These findings suggest a complex interaction between the vascular endothelium, iNOS, and NO in modulating contractile function during viral infections. Neutralizing NO with Tiron eradicates variations in contractile responses to PE between aortic rings from Mock-treated and CHIKV-infected mice. Thus, both iNOS and NO emerge as potential therapeutic targets to modulate vascular responses during viral infections. Collectively, these studies suggest that CHIKV and other viruses interfere with blood pressure and vascular tone regulation and point to potential therapeutic targets for managing vascular complications during viral outbreaks.

Despite some shortcomings, this work offers valuable insights into how endothelial dysfunction and vascular function are affected by Chikungunya virus (CHIKV) infection. The 48 h post-infection length may not accurately reflect the long-term effects of CHIKV on vascular functioning, and longer-term studies may reveal persistent vascular changes caused by the virus. This investigation’s emphasis on nitric oxide (NO) production, ROS, and NF-κB signaling may underscore the comprehension of other molecular pathways that may contribute to vascular dysfunction. Furthermore, additional studies are needed to determine the precise processes by which CHIKV infection impairs vascular function, to enhance clinical care for affected individuals, and to prevent or reduce cardiovascular difficulties caused by Chikungunya.

## 5. Conclusions

This study highlights that CHIKV infection leads to vascular alterations linked to increased formation of NO and ROS, and activation of the NF-κB pathway. Additional studies are needed to discover the precise mechanisms whereby CHIKV infection affects vascular function and to improve clinical care for affected individuals, as well as to prevent or mitigate cardiovascular issues caused by Chikungunya.

## Figures and Tables

**Table 1:** pEC_50_ values for PE and ACh in aortas from WT_Mock mice and WT_ CHIKV mice at times of 24, 48, and 72 h after infection/vehicle injection.

	24 h		48 h		72 h	
	PE (% KCl)	ACh	PE (% KCl)	ACh	PE (% KCl)	ACh
**WT_Mock**	7.05 ± 0.06	7.61 ± 0.07	7.08 ± 0.09	7.82 ± 0.06	7.09 ± 0.09	7.82 ± 0.03
**WT_CHIKV**	7.02 ± 0.15	7.68 ± 0.06	6.79 ± 0.09 *	7.68 ± 0.10	7.00 ± 0.08 *	7.80 ± 0.09

Data represent the mean ± S.E.M (*n* = 5–7 mice per group). Two-way ANOVA: * *p* < 0.05 vs. WT_Mock. Emax: response, pEC_50_: negative logarithm of the EC_50_, PE: phenylephrine, ACh: acetylcholine, WT: wild type.

**Table 2:** pEC_50_ values for PE in aortas from WT mice incubated with L-NAME, 1400 W, Tiron, or vehicle, and aortas from iNOS^−/−^ mice.

	Vehicle	L-NAME	1400W	Tiron
**WT_Mock**	7.05 ± 0.06	7.15 ± 0.09 *	7.07 ± 0.07 *	7.07 ± 0.18
**WT_CHIKV**	7.02 ± 0.15 *	7.12 ± 0.14 *^#^	7.31 ± 0.04 *^#^	7.15 ± 0.24 ^#^
**iNOS^–/–^_Mock**	7.37 ± 0.05 *	-	-	-
**iNOS^–/–^_CHIKV**	7.42 ± 0.05 *	-	-	-

Data represent the mean ± S.E.M (n = 4–10 mice per group). Two-way ANOVA: * *p* < 0.05 vs. Mock. ^#^ *p* < 0.05 vs. CHIKV. Emax: maximal response, pEC_50_: negative logarithm of the EC_50_, PE: phenylephrine, ACh: acetylcholine, WT: wild type; iNOS^−/−^: iNOS *knockout*.

## Data Availability

The data presented in this study are available in this article.

## Supplementary Materials

The following supporting information can be downloaded at https://www.mdpi.com/article/10.3390/cells13211770/s1, Table S1: Body mass (g) of WT and iNOS^−/−^ mice infused with CHIKV or Mock.
