# Supplementary material for: Acute Chikungunya Infection Induces Vascular Dysfunction by Directly Disrupting Redox Signaling in Endothelial Cells
Source: Cells. 2024 Oct 25;13(21):1770. doi: 10.3390/cells13211770 (PMC11544861; doi:10.3390/cells13211770)
Supplement: Supplementary file 1 [file cells-13-01770-s001.zip › cells-3221997-supplementary.pdf]

Article

Acute Chikungunya infection induces vascular dysfunction by directly disrupting redox signaling in endothelial cells

José Teles de Oliveira-Neto [1,*], Juliano de P. Souza [2], Daniel Rodrigues [1], Mirele R. Machado [1], Juliano V. Alves [1,3], Paula R. Barros [1,4], Alecsander F. Bressan [5], Josiane F. Silva [1], Tiago J. Costa [4], Rafael M. Costa [1,3], Daniella Bonaventura [6], Eurico de Arruda-Neto [2], Rita C. Tostes [1] and Emiliana P. Abrão [1,7]

Supplementary material

Table S1. Body mass (g) of WT and iNOS$^{-/-}$ mice infused with CHIKV or Mock

	WT_Mock	WT_CHIKV	iNOS$^{-/-}$_Mock	iNOS$^{-/-}$_CHIKV
**Before infection**	20.83 ± 0.33	20.13 ± 0.52	21.23 ± 1.10	22.1 ± 0.55
**After infection**	20.41 ± 0.32	19.33 ± 0.38	21.5 ± 1.00	21.96 ± 0.80

Data (body mass in g) represent the mean ± S.E.M (n= 3-6 mice per group). Two-way ANOVA: * $p<0.05$ *vs.* respective Mock group.
